# Dynamics of the Third Wave of COVID-19 from the Perspective of the Emergency Department in a Large Regional Hospital—Single Center Observational Study

**DOI:** 10.3390/healthcare10010018

**Published:** 2021-12-23

**Authors:** Tomasz Kłosiewicz, Weronika Szkudlarek, Magdalena Węglewska, Patryk Konieczka, Radosław Zalewski, Roland Podlewski, Anna Sowińska, Mateusz Puślecki

**Affiliations:** 1 Department of Medical Rescue, Faculty of Health Sciences, Poznan University of Medical Sciences, 60-608 Poznan, Poland; zalewski.rad@gmail.com (R.Z.); rpodlew@ump.edu.pl (R.P.); mateuszpuslecki@o2.pl (M.P.); 2 Students’ Scientific Circle of Emergency Medicine, Department of Medical Rescue, Faculty of Health Sciences, Poznan University of Medical Sciences, 60-608 Poznan, Poland; wer.szkudlarek@gmail.com (W.S.); magda74747@wp.pl (M.W.); 3 Department of Emergency Medicine, Faculty of Health Sciences, Poznan University of Medical Sciences, 60-608 Poznan, Poland; patryk@konieczka.net; 4 Department of Computer Science and Statistics, Poznan University of Medical Sciences, 60-608 Poznan, Poland; ania@ump.edu.pl; 5 Department of Cardiac Surgery and Transplantology, Medical Faculty, Poznan University of Medical Sciences, 61-848 Poznan, Poland

**Keywords:** COVID-19, pandemic, emergency department, decision making process, fast-track

## Abstract

Background: The outbreak of the Coronavirus Disease 2019 (COVID-19) pandemic has caused many significant social and economic changes. The consecutive waves of the epidemic in various countries have had dissimilar courses depending on the methods used to combat it. The aim of this study was to determine the dynamics of the third wave of COVID-19 from the perspective of emergency departments (ED). Methods: This was a retrospective review of medical records from ED. The authors have identified the most frequent symptoms. Prognostic factors have been chosen—prognostic scales, length of stay (LOS)—and a number of resources required have been calculated. Results: As the time passed, there were fewer patients and they presented mild symptoms. A statistically significant difference was observed in the median of blood oxygenation measurement (*p* = 0.00009), CRP level (*p* = 0.0016), and admission rate. Patients admitted to the hospital required more resources at ED. LOS was shorter in patients discharged home (*p* < 0.0001). Conclusions: The blood oxygen saturation (SPO2) and CPR levels can be helpful in decision-making regarding medical treatment. The fast-track for patients in good clinical condition may shorten the duration of stay in ED, and reduce the number of required resources.

## 1. Introduction

The rapid spread of coronavirus was noticed in December 2019 in China. The disease mainly causes acute aspiratory failure. The World Health Organization (WHO) used the name of Coronavirus Disease 2019 (COVID-19) to identify the virus that quickly spread all over the world, which, in turn, caused WHO to announce the pandemic [1,2]. Patients infected with COVID-19 present a wide range of clinical conditions: an asymptomatic infection, a mild course of infection, or a severe viral pneumonia leading to acute respiratory failure followed by death [3]. Emergency departments (ED) are often the place of the first contact between a patient and a medical professional. These are places where not only patients with COVID-19, but also those with other medical problems are attended to without prior appointment. Diagnostics and therapies of varying degrees of complexity are performed. This makes the work in an ED a major organizational challenge; it requires the appropriate allocation of human and material resources. In this COVID-19 era, frontline medical staff in ED are facing even more new challenges to diagnose and treat patients. The ongoing COVID-19 pandemic has had a huge, devastating impact on healthcare organizations. It has also affected the increased mortality of non-COVID-19-related illnesses [4]. ED should focus on early recognition, immediate isolation, as well as appropriate infection prevention with care taken to optimize supportive care. This refers not only to patients with suspected COVID-19, but also to doctors and nurses working at the ED units, as they are at a significant risk of accidental infection. Appropriate infection prevention should be strict, and include all PPE instructions. [5]. With the presence of well-known overcrowding problems and limited resources, forecasting patient influx for ED care is a key solution to maintaining fluent work conditions. This overcrowding is manifested mainly by a prolonged waiting time and length of stay [6,7]. The ability to perform smooth reorganization between periods with different inflows of COVID-19 patients may be important, especially for facilities with a small number of beds within ED, and limited staff.

The objective of this study was to determine the course of the third wave of COVID-19 based on observations of symptoms, severity, and workload in the perspective of the ED in order to better understand and, hopefully, predict the impact of future pandemic waves on the healthcare system. It also aimed at determining if a dynamic increase in COVID-19 incidence rate coincides with a drop of severity of the patients’ symptoms severity on presentation.

## 2. Materials and Methods

### 2.1. Study Design

The study was designed as a retrospective review of medical records. The study was performed in ED at Hipolit Cegielski Medical Center, Poznań, Poland. Records from the hospital information system were used. The data were prepared by authorized professionals to ensure the protection of personal information. According to local regulations, this study did not meet the criteria of a medical experiment, and the approval of the Bioethics Committee was not required.

### 2.2. Study Protocol

The subjects of interest were individuals diagnosed with COVID-19 (according to ICD-10 classification U07.1). All patients with this diagnosis were included in the group. Individuals younger than 18 years old, and those admitted due to trauma were excluded, as the occurrence of trauma could not be related to COVID-19 in any direct way.

### 2.3. Emergency Department

This department was dedicated to adults only. Nevertheless, if a pediatric patient came in, physical examination was performed by a physician and, if necessary, the patient was transported to a children’s hospital. The department was staffed with emergency medicine specialists and residents. The Manchester Triage System was used to select priorities of admission and treatment. The system was not modified or adapted for COVID-19. At the triage point, a paramedic measured critical vitals: heart rate (HR), respiratory rate (RR), non-invasive blood pressure (NIBP), hemoglobin oxygen saturation (SpO2), Glasgow Coma Scale (GCS), temperature. If a patient presented any signs of infection, Abbott’s antigen test (Abbott Rapid Diagnostic Jena GmbH, Jena, Germany), which detects viral proteins in the nasopharynx, was used. The test was performed by experienced and trained nurses, or paramedics working in the ED. In case of an unclear result or high clinical suspicion of infection with a negative antigen test, the RT-PCR method was used as appropriate to detect viral mRNA. The result was entered into a nationwide registry. A patient with a positive test result was referred to the isolation area. Based on the examination in triage, a further pathway for the patient was determined. Those in a good condition who did not require oxygen therapy were referred to the waiting room in the isolation area. Those requiring oxygen therapy were directed to the observation room in the isolation area. There were incidents where a COVID-19 patient was referred from triage to the examination area without a test. This was a group of patients who did not present symptoms of COVID-19 infection. In this case, the test was performed on a physician’s order. The patient was immediately transferred to the isolation area, and appropriate disinfection procedures were implemented. All personnel were required to wear personal protective equipment (gloves and surgical mask) and maintain social distance whenever possible. Personnel working in the infectious area were equipped with safety goggles, a FFP3 respirator mask, and a full protective suit complying with the European standard EN 14126.

### 2.4. Selection of Variables

Based on the review performed by Grant et al. [8], the most common symptoms were defined. These were further divided into four groups: systemic (fever, myalgia, fatigue, near fever), respiratory (dyspnea, cough, chest pain), gastrointestinal (abdominal pain, diarrhea, nausea/vomiting, anosmia/ageusia), ear–nose–throat (sore throat, vertigo, rhinitis). Other symptoms were also reported in the analyzed records, but their frequency was sporadic, and they were not included in further analyses.

Prognostic factors were defined based on the review of Izcovich et al. [9]. Their identification indicates the severity of the disease. Among these proposed by Izcovich, the authors selected the level of WBC (white blood cells), PLT (platelet count), CREA (creatinine in serum), and CRP (C-reactive protein). Other biochemical tests were discarded because they were not run on every patient. Parameters such as D-dimers, LDH (lactate dehydrogenase), or liver enzymes were evaluated only in reasonable cases.

Two prognostic scales were also calculated based on the selected parameters:

Quick Sequential Organ Failure Assessment Score for Sepsis (qSOFA), which identifies high-risk patients for in-hospital mortality with suspected infection outside the intensive care unit (ICU). This scale includes mental status, RR, and systolic blood pressure.

Quick COVID-19 Severity Index (qCSI). This scale is to stratify the risk of critical illness at 24 h, defined by oxygen requirement (>10 L/min by low-flow device, high-flow device, non-invasive, or invasive ventilation) or death. This scale includes RR, SpO2, and oxygen flow rate.

Resources were defined as laboratory tests (blood, urine); electrocardiogram; radiographs; computed tomography; point of care ultrasound; intravenous fluids; intravenous, intramuscular, or nebulized medications; simple procedure (urinary catheter, oxygen supplementation). Complex procedures, such as endotracheal intubation, procedural analgosedation, blood transfusion, and mechanical ventilation, were counted as two resources.

Length of stay (LOS) was defined as the time from when a patient was admitted by a physician until the time of the discharge or admission to a ward. The time when patients had been waiting for admission after triage was not calculated.

### 2.5. Statistical Analysis

First, the quantitative variables were checked for normality with the use of the Shapiro–Wilk W test. As none of the analyzed parameters met the criteria of a normal distribution, they were presented as median (interquartile range). The categorical variables were expressed as the numbers (n) with percentages (%). For statistical analysis, Kruskall–Wallis and Mann–Whitney tests were used as appropriate. Spearman’s correlation rank was used to assess a correlation between variables. A p-value less than 0.05 was considered significant. The analysis was performed using the Statistica 12 software (Tibco Inc., Tulsa, OK, USA).

## 3. Results

### 3.1. Study Group

During the study period, a total number of 862 patients diagnosed with COVID-19 were admitted to our department. In this group, five patients were excluded due to the lack of data, and four due to an age <18 years. Among them, there was also a group of 75 patients admitted directly to the department of COVID-19 treatment, whose presence in the ED was caused only by the need to register in the hospital information system. These individuals were not examined by a physician, but only passed through the ED. Finally, 778 cases were included in the analysis.

**Figure 1:**
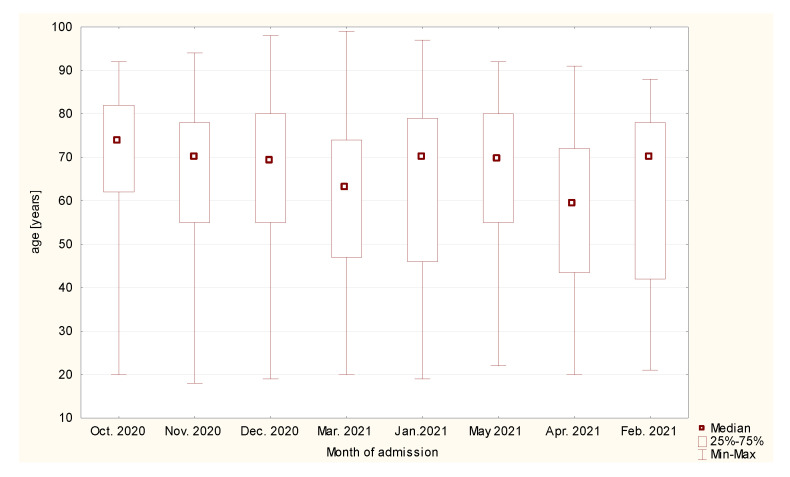
Median age depending on the month of admission.

There were 371 (47.7%) women, and 406 (52.25%) men in the study group. The median age was 68 (50–78) years. Most of patients were aged 65 to 79 years old. This group constituted 287 patients (36.94%). There were 167 patients older than 80 years old (21.49%), 163 patients aged 45 to 64 years old (20.98%), and 133 individuals aged 25 to 44 years old (17.12%). The least common group were young adults between 18 and 24 years old (n = 27, 3.47%). The median age depending on the amount of admission is presented in Figure 1.

**Figure 2:**
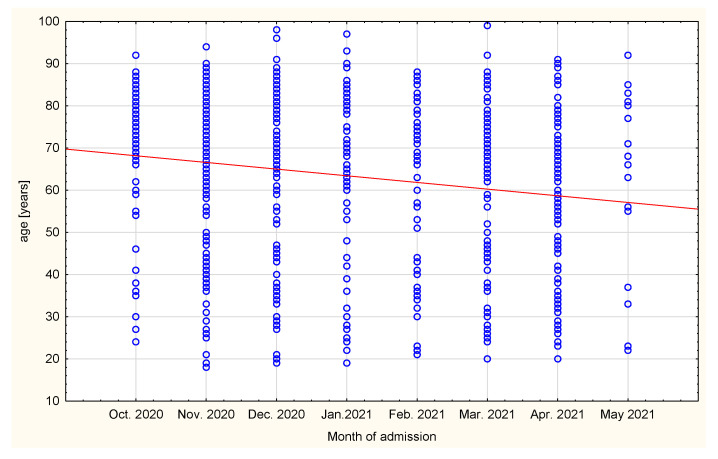
Correlation between age of the patients and month of admission.

A statistically significant difference was found in month of admission and age of the patients (r = −0.1488; *p* = 0.00003, Figure 2). At the beginning of the third wave, more older patients were admitted to ED (74 (66–82) vs. 70 (42–78) years old).

One dot represents one patient.

Four-hundred and thirty-seven (56.24%) patients with positive test results were discharged after medical consultation. Three-hundred and twenty-nine (42.34%) were admitted to a unit other than the ICU. Seven patients (0.90%) died in the ED due to acute respiratory distress followed by cardiac arrest. Four patients (0.51%) were admitted to the ICU. There were 211 patients admitted in November, 129 admitted in December, 109 in March, 108 in April, 96 in January, 78 in October, 68 in February, and 58 in May.

### 3.2. Symptoms

The predominant symptoms were dyspnea, fever, cough, and fatigue. Regardless of the month, more than 1/4 of all patients presented at least one of these symptoms. Other symptoms were present in less than 10 percent of cases. Systemic symptoms were present in almost every patient. For the remaining individuals, respiratory symptoms were the most prevalent (Table 1). Although there was a decrease in the number of the four most common symptoms (dyspnea, fever, fatigue, cough), as well as anosmia and ageusia, only in the case of dyspnea was the difference proven to be statistically significant (*p* = 0.006149).

### 3.3. Prognostic Factors

The median prognostic scales were as follows: qSOFA 0 (0-1), qCSI 10 [7,8,9,10], SpO2 94% (88–96), WBC 7.24 (5.27–10.5), PLT 197 (147–278), CRP 89 (34–163), and CREA 1.04 (0.82–1.43). There was no statistical relationship between qCSI (*p* = 0.1068) or qSOFA (*p* = 0.7854) scores depending on the month of patient admission to the ED. However, there was a statistically significant difference in the median of first blood oxygenation measurement (KW-H(7;675) = 30.0383; *p* = 0.00009), CRP level (KW-H(7;455) = 23.1584; *p* = 0.0016), and PLT (KW-H(7;476) = 19.263; *p* = 0.0074). November was a month with a wider scatter of the results in all analyzed parameters. 

Table 2 presents median values of each prognostic parameter divided into patients admitted to hospital and those discharged home. Statistical tests showed significant difference in hemoglobin saturation (*p* < 0.0001), WBC (*p* < 0.0001), PLT (*p* = 0.001), CRP (*p* < 0.0001), and CREA (*p* < 0.0001). However, there was no difference in qCSI and qSOFA scales.

**Figure 3:**
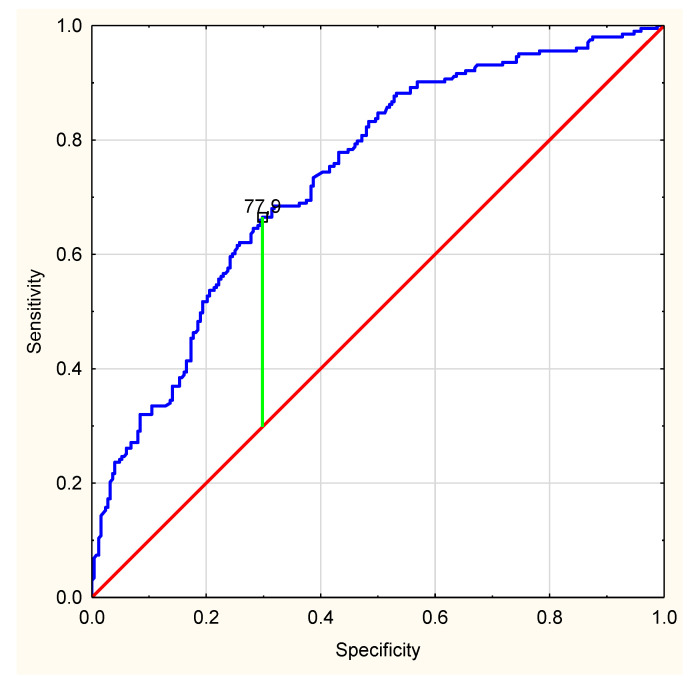
—ROC curve for C-protein level. Line of no-discrimination is marked red. Serum C-reactive protein level is marked blue.

The ROC curve for CRP level shows that there is a cut-off point of 77.9 mg/L. Patients with CRP above this level are more likely to be admitted than discharged (AUC 0.738, *p* < 0.0001) (Figure 3).

### 3.4. Resources

Patients required three (1–4) resources during their stay in the ED. Patients who died required the greatest number of resources (4 (3–7)), followed by those admitted to the ICU (4 (2–5)), and then those admitted to the non-ICU (3 (3,4)). Patients discharged home required the least resources (2 (0–3)). Only the latter two groups were included in the statistical analysis because of the very large difference in group sizes in the remaining groups. It was shown that the difference in the number of resources used to treat patients admitted to hospital and discharged home was statistically significant (*p* < 0.0001).

### 3.5. Length of Stay

**Figure 4:**
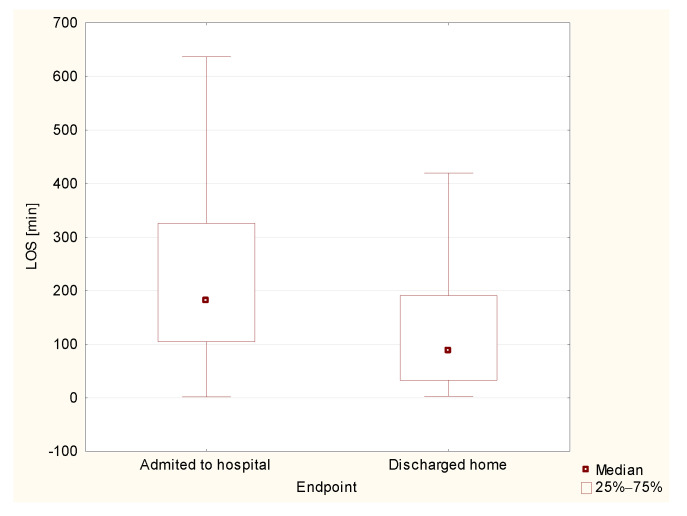
Median length of stay in the emergency department in the group of patients admitted to the hospital and those discharged home.

The median LOS in the ED was 134 min (47–251). The shortest length of stay was 2 min, whereas the longest lasted 3063 min. This parameter varied between groups. The lowest median LOS was in the group of those discharged home (89 min (10–1470). This was followed by those admitted to the intensive care unit (140 min (37–532)), followed by those admitted to non-ICU ward (183 min (362–3063)). The highest median length of stay concerned deceased patients—293 min 965–867). In the analysis of statistical significance of differences in LOS, the groups of deceased patients and patients admitted to the intensive care unit were excluded due to a very small size of these groups, as in previous statistics. The analysis showed statistically significant differences in length of stay between the groups of patients discharged home and those admitted to the hospital (*p* < 0.0001, Figure 4).

The longest LOS was in October (342 min (157–922)), then May (176 min (121–337)), November (149 min (65–256)), March (129 min (24–226)), December (127 min (74–243)), April (115 min (35–246)), and January (102 min (34–183)). The shortest LOS was observed in February (79 min (26–173)). The difference between November and all other months was statistically significant (for each pair *p* < 0.0001). Also, the difference between November and January (*p* = 0.028364) and February (*p* = 0.029543) was significant, although the length of stay was comparable.

## 4. Discussion

There were three hospitals with ED in Poznań, Poland. One of the hospitals was converted as a reference hospital only for patients diagnosed with COVID-19. This hospital did not admit patients without a diagnosis of this disease. Thus, the ED where the study was conducted was one of the two ED operating in the city of Poznań at the time of analysis. The operating area of this department doubled, as it was in a hospital neighboring a COVID-19 hospital. An isolation area was set up within the department for patients with a positive test result. In addition, there was one room for patients who were suspected of COVID-19 to wait for their test results. The isolation area consisted of an observation area and a waiting room. The observation area was equipped with three beds, each with access to oxygen. Basic medical equipment was available in the room. Also, resuscitation equipment to ensure advanced life support and initial intensive care was available. The design of the area allowed for the one-way passage of the staff. There was a separate exit to the ambulance bay. A dedicated nursing staff worked in the isolation area. A physician was called at a nurse request. If a new patient was admitted, a physician examined this patient in an order established by a triage paramedic. Patients not requiring oxygen therapy were examined by a physician. Then, a consultation was provided, and the patient was discharged home or referred to the observation area for further diagnosis or treatment. With longer LOS, the chance of cross-infection increases, and the health workers working in ED will be at risk. Therefore, activities should be made to shorten the LOS for patients with signs and symptoms of respiratory tract infection. The efficacy and legitimacy of a fast-track for minor medial cases were previously proven by other authors [10,11,12]. This approach can significantly reduce the length of stay in the ED. However, this approach requires a robust patient selection procedure based on the evidence-based medicine. The use of predictive scales, ideally based on parameters or markers measured in the ED, without the need for laboratory or radiodiagnostic involvement could be an important component of the fast-track for COVID-19 patients presenting to the ED.

About 80% of COVID-19 patients have mild or moderate disease, whereas approximately 15% of patients develop severe disease that requires oxygen support, and 5% have critical disease with complications such as respiratory failure or severe sepsis [13]. The primary goal of an ED is to triage patients whose current or developing symptoms may lead to deterioration. The role of an emergency physician is to vigilantly observe symptoms. Biochemical parameters or basic vital signs may be helpful in making decisions regarding discharge home or admission to a hospital. Treatment of patients with COVID-19 is difficult. This is primarily because of the dynamics of the disease itself. In a patient presenting to the ED, a physician looks mainly for symptoms of respiratory failure during the examination. On this basis, a decision is made whether to treat the patient in the hospital or at home. Hensgens suggested that ED screening protocols should include non-specific complaints, particularly in older patients, to improve early identification and proper segregation [14].

Over the past year, new scales and ratios were developed to support decision-making, and assess the risk of severe exacerbation [15]. However, these are not tools on the basis of which a patient can be safely discharged home. In the conditions studied, neither qSOFA nor qCSI were a useful tool to estimate further decision. Other authors also suggest that these scales do not have proper efficacy to predict death, ICU admission, and disease severity of COVID-19 [16]. In our analysis, a different level of selected biochemical parameters in patients admitted to hospital and those discharged home has been presented. Nevertheless, it should be noted that the median results of WBC, PLT, as well as CREA, although they varied in both groups, remained within normal ranges. Therefore, it seems that their routine evaluation is not necessary. Thus, two parameters seem to be important: CRP and SpO2. CRP values were almost three times different in both groups. The level of plasma CRP is positively correlated to the severity of COVID-19 pneumonia lung lesions. CRP testing may be useful as an earlier indicator for severe illness, and may help physicians to stratify patients for intensive care unit transfer [17,18]. CRP level was also positively correlated with the diameter of lung lesion. Moreover, CRP concentration higher than 32.5 mg/L showed high rates of sensitivity to detect patients at risk for respiratory failure [19]. Higher levels at presentation might indicate impending clinical deterioration and the need for mechanical ventilation [20]. Our results suggest that patients with CRP level > 77.9 mg/L were more likely to be admitted to hospital. Therefore, we suggest to pay special attention to this group of patients when considering further decisions. Akhavan et al. [21] reported similar values of oxygen saturation in admitted and discharged patients (89% vs. 96%). The authors concluded, however, that this parameter may be helpful in assessing the need of oxygen supplementation. It is less useful for identifying which patients may deteriorate clinically in the days after ED discharge, requiring subsequent hospitalization. In the presented study, these two were the parameters that showed the greatest differences when comparing November to other months. Blood saturation is a better predictor of deterioration in COVID-19. This is related to the “silent hypoxia” phenomenon [22,23]. The patient does not experience dyspnea for a long time, despite the developing destructive process in the lungs. The parameters we analyzed in the study are also the basis of the PANDEMYC scale. This interesting tool was proposed and validated by Torres-Macho and colleagues [24]. In their wide-ranging and pioneering investigation, the authors estimated mortality risk based on the basic parameters of blood count, CRP, creatinine, saturation, sodium, age, and smoking status. Their approach supports our results on the significance of CRP and saturation in the decision-making process. In the future, it would be interesting to define a cut-off point for hospital admission. In this way, the PANDEMYC scale could be a real support for ED physicians.

Interestingly, in the last months of the pandemic, the number of patients complaining of dyspnea, cough, fever, or anosmia decreased. It is likely that patients were presenting to the hospital earlier than in previous months. This may be the result of a greater public awareness of the symptoms, or the fear of getting sick. Patient outcomes are worse when the second and higher stages of the disease occur.

Understanding the trend of patient flow in an emergency department is important to ensure the best possible management. According to the results presented in this study, patients’ length of stay in the ED was the longest in October. In the authors’ opinion, the reason for this was the overall large increase in the number of cases, and the general overload of the system. There were logistical problems regarding the ability to transport and admit patients to COVID-19-dedicated hospitals. As a result, patients waited a long time to be transferred to another hospital or to COVID-19 treatment units. Patients admitted to the hospital usually required three or four resources, more than those discharged home. Staff efficiency was further limited by the need for increased epidemiological rules and protective equipment. It should be noted that in December, a field hospital was established at the Poznan International Fair. This facility received many patients, relieving the previously overloaded wards. Such hospitals allow effective treatment of patients who do not require advanced therapies. At the same time, they concentrate all resources necessary for COVID-19 treatment in one place [25].

The authors are aware of limitations of this paper. The number of individuals in the study group was relatively small. There are differences in group sizes between months. There is, therefore, a risk of statistical error. This is due to the characteristics of this wave of infections, and the fluctuating variability in the number of cases. The results are based on data from a single hospital. There was no prepared form based on which physicians performed the anamnesis and further diagnosis. Moreover, the hospital admission policies may differ, and, thus, the results cannot be generalized to other hospitals or the general population. Also, further treatment was physician-dependent. Therefore, the analyzed group cannot be a national representative. The results may be different in other cities. Many patients had comorbidities. These may have affected the results of particular laboratory tests. In this situation, it would be beneficial to divide patients using the Charlson Comorbidity Index. This scale predicts 10-year survival in patients with multiple comorbidities. However, in this study, we did not analyze chronic diseases. In addition, in many cases, working in isolation, and overcrowding made it difficult to keep detailed records, which were often completed in the destination ward.

## 5. Conclusions

The largest number of patients with COVID-19 symptoms were admitted to ED at the beginning of the third wave (October 2020–May 2021). As time progressed, patients presenting with milder symptoms were observed. ED should be prepared for an increased influx of patients at the beginning of the wave. This is the period that may require reorganization of the workplace to the greatest extent. It is worth considering a fast-track for patients in a good clinical condition to reduce the length of stay in the ED, the risk of accidental infection in healthcare providers, and the number of resources required. CRP levels greater than 77.9 mg/L may be helpful in the decision-making process. Being on the threshold of the fourth wave of the COVID-19 pandemic, taking into account the changing profile of patients and preventive vaccinations, further studies are necessary to determine the decision predictors.

## Figures and Tables

**Table 1:** The most common symptoms of COVID-19 in the analyzed group.

Symptom	*n*	%
**Systemic**	740	95.1
*Fever*	346	44.5
*Fatigue*	255	32.8
*Myalgia or arthralgia*	50	6.4
*Near fever*	89	11.4
**Respiratory**	663	85.2
*Dyspnea*	318	40.9
*Cough*	272	35.0
*Chest pain*	73	9.4
**Ear-nose-throat**	101	13.0
*Sore throat*	17	2.2
*Rhinitis*	17	2.2
*Vertigo*	17	2.2
**Gastrointestinal**	167	21.5
*Anosmia or ageusia*	50	6.4
*Diarrhea*	48	6.2
*Vomiting/nausea*	58	7.4
*Abdominal pain*	61	7.8

**Table 2:** Prognostic factors in patients admitted to hospital and discharged home.

	Admission		Discharged		
Parameter	Median	IOR	Median	IOR	*p*-Value
qSOFA	0	0–1	0	0–0	0.7854
qCSI	10	44,476	7	7–10	0.1068
WBC	8.32	6.10–11.30	6.23	4.77–8.60	0.000000
PLT	217	154–287	183	141–244	0.001139
CRP	121	65–194	51	15–103	0.000000
CREA	1.20	0.85–1.82	0.95	0.78–1.18	0.000001
SpO2	89	82–94	95	93–97	0.000000

Abbreviations: qSOFA—Quick Sepsis Related Organ Failure Assessment; qCSI—Quick COVID-19 Severity Index; WBC—white blood cells; PLT—platelet count; CRP—C-reactive protein; CREA—creatinine in serum; SpO2—hemoglobin oxygen saturation; IQR—interquartile range.

## Data Availability

Data are available from the corresponding author on reasonable request.
